# Scale development and validation of perimenopausal women disability index in the workplace

**DOI:** 10.1265/ehpm.23-00239

**Published:** 2024-02-02

**Authors:** Kyoko Nomura, Kisho Shimizu, Fumiaki Taka, Melanie Griffith-Quintyne, Miho Iida

**Affiliations:** 1 Department of Environmental Health Science and Public Health, Akita University Graduate School of Medicine, Akita, Japan; 2 Faculty of Sociology, Toyo University, Tokyo, Japan; 3 Department of Preventive Medicine and Public Health, Keio University School of Medicine, Tokyo, Japan

**Keywords:** Disability index, Internet survey, Perimenopausal period, Productivity, Working women

## Abstract

**Background:**

Menopausal disorders include obscure symptomatology that greatly reduce work productivity among female workers. Quantifying the impact of menopause-related symptoms on work productivity is very difficult because no such guidelines exist to date. We aimed to develop a scale of overall health status for working women in the perimenopausal period.

**Methods:**

In September, 2021, we conducted an Internet web survey which included 3,645 female workers aged 45–56 years in perimenopausal period. We asked the participants to answer 76 items relevant to menopausal symptomatology, that were created for this study and performed exploratory and confirmatory factor analyses for the scale development. Cronbach’s alpha, receiver operating characteristic analysis, and logistic regression analysis were used to verify the developed scale.

**Results:**

Approximately 85% participants did not have menstruation or disrupted cycles. Explanatory factor analysis using the maximum likelihood method and Promax rotation identified 21 items with a four-factor structure: psychological symptoms (8 items, α = 0.96); physiological symptoms (6 items, alpha = 0.87); sleep difficulty (4 items, alpha = 0.92); human relationship (3 items, alpha = 0.92). Confirmatory factor analyses found excellent model fit for the four-factor model (RMSR = 0.079; TLI = 0.929; CFI = 0.938). Criterion and concurrent validity were confirmed with high correlation coefficients between each of the four factors, previously validated menopausal symptom questionnaire, and Copenhagen Burnout Inventory scales, respectively (all ps < 0.0001). The developed scale was able to predict absenteeism with 78% sensitivity, 58% specificity, and an AUC of 0.727 (95%CI: 0.696–0.757). Higher scores of each factor as well as total score of the scale were more likely to be associated with work absence experience due to menopause-related symptoms even after adjusting for Copenhagen Burnout Inventory subscales (all ps < 0.0001).

**Conclusion:**

We found that the developed scale has high validity and reliability and could be a significant indicator of absenteeism for working women in perimenopausal period.

**Supplementary information:**

The online version contains supplementary material available at https://doi.org/10.1265/ehpm.23-00239.

## 1. Introduction

The perimenopausal period, which typically begins during the mid-to-late 40s and ends within 4 to 7 years [1], with irregularities in cycle length, refers to the interval immediately preceding menopause (i.e., cessation of menses) [2]. Menopause is not a disease but a syndrome with various manifestations, including psychological symptoms (i.e., depression, insomnia), vasomotor symptoms (i.e., hot flushes and sweating), and physical symptoms (i.e., muscle pain and fatigue) [3, 4]. Among these, perimenopausal depression is characterized as a mild mood disorder associated with anger, irritability, and paranoia, but may significantly affect the quality of daily life, including work efficiency, and human relationship [5, 6]. In a systematic review, longer estrogen exposure periods were reported to protect women from depression [7]. This indicates that perimenopausal women may be at greater risk for depression due to a sudden decrease of estrogen level. In addition, aging causes dysregulation of gonadal steroids in the hypothalamic–pituitary–adrenal axis that may alter the levels of stress hormones. Apparently, a complex interplay exists between hormonal vulnerability, available psychosocial resources (coping skills and social support), overall well-being (exercise and other lifestyle factors), and demands on coping resources (stressful life events) [8]. Recent negative life events, a history of depression, and severity of somatic symptoms of perimenopause were reported to predict unique variances in depression scores [9]. However, research on the role of psychosocial factors in increasing the stress level in women and how it relates to the etiology of reproductive mood disorders is scarce [10]. This may be because disclosure of menstrual symptoms or reasons for sickness absence due to menstrual symptoms is low [11], because it is a personal or embarrassing topic for women.

A previous study reported that 80% of middle-aged women aged 45–56 years have at least one menopause-related symptom while only one-forth had sought healthcare [12]. Such menstrual symptomatology has a great impact on work productivity. In fact, a large study on 32,748 women aged 15–45 years showed that 80.7% reported presenteeism whereas 13% reported absenteeism, suggesting significant influence on work engagement [11]. As these gender-related health issues are often not recognized, women are often mistreated and harassed in the workplace [13]. It is obvious that menopause severely deteriorates the quality of daily life and work productivity [14] and eventually leads to an economic burden on society [15]. There have been no guidelines that can be used in labor management, to measure comprehensive health status of working women in perimenopausal period. In clinical practice, the Kupperman Index [16] was created in 1953 worldwide as a patient-reported outcome for menopause, and the Japan Society of Obstetrics and Gynecology created the Menopausal Symptom Questionnaire (MSQ) [17] in 2003 in Japan. Although these previously invented indicators cover the various physical and psychological symptoms commonly seen in menopause, they do not take into account the social aspects such as human relationships at work, among family members, friends, and acquaintances that menopausal women often face. Research has shown that individuals exposed to psychosocial stressors are more likely to develop mental illness in the presence of menopausal hormone fluctuations, suggesting the importance of considering social factors when evaluating disorders of menopausal women [5, 6, 18]. Hence, the purpose of this study was to develop a validated scale that can be used to objectively assess the overall health status of female workers in the perimenopausal period.

## 2. Methods

### 2.1 Original items for scale development of perimenopausal women disability index

A task team comprising an industrial physician, gynecologist, psychosomatic specialist, and public health practitioner identified 76 items relevant to menopausal symptomatology associated with physical, psychological, and human relationship aspects in the workplace and daily life referring to previous menopause index (Appendix).

The participants were posed the question “During the period when your menstrual cycle began to change (short, low volume, irregular, etc.) or in the years before and after menopause (if you are not sure, please answer with your current symptoms), how often did you have any of the following symptoms?” and asked to respond for each item on the following 8-point scale: 0 (none), 1 (almost none), 2 (1 or less per month), 3 (twice or more per month), 4 (about once per week), 5 (2–3 times per week), 6 (4–6 times per week), and 7 (every day).

### 2.2 Study participants

This study was conducted by an Internet research company (GMO Research, Inc., Tokyo, Japan). The GMO Research owned their panel (closed registry with internet recruitment), that included total 117,870 women (72,553 in their 40’s and 38,985 in their 50’s). The inclusion criteria were female workers aged 45–56, who resided in Japan at the time of investigation, regardless of whether they worked full-time or part-time, and regardless of ethnic background. Exclusion criteria were female workers outside the 45–56 age group, male workers, and female workers who were not working at the time of investigation. The age range of the recruits was based on a previous report that the median age of menopause in Japan is 50.5 years, with a range of 45–56 years [19]. The GMO Research declared that the company is fully aware of its social mission regarding the protection of personal information, and complies with laws and regulations regarding the protection of the rights of individuals and personal information. The informed consent was obtained by this company and the data availability to the third party was also included in the consent. This internet research company distributed questionnaires between September 27 and 30, 2021, to registered 15,279 survey participants who have agreed to the terms and conditions of the survey on the premise that they would cooperate with the survey request. Among those, 3951 indicated their willingness to participate in the survey; however, 3,645 actually completed the self-administered questionnaire.

The protocol of this study was approved by ethical committee of Akita University, School of Medicine (No. 2712, approval date, July 7^th^, 2021).

### 2.3 Questionnaire

The items included age, height, weight, marital status, number of children, household income (>8 million Japanese Yen, JPY/6–8 million JPY/4–6 million JPY/2–4 million JPY/<2 million JPY), educational attainment (elementary/junior/high school/2-year college/university or graduate school), occupation (the Japan Standard Occupational Classification; clerical workers/service workers/professional and engineering workers/workers not classifiable by occupation/sales workers/manufacturing process workers/administrative and managerial workers/transport and machine operation workers/construction and mining workers/agriculture, forestry and fishery workers/carrying, cleaning, packaging, and related workers/security workers), industry (the Japan Standard Industrial Classification; medical, health care and welfare/manufacturing/compound services/wholesale and retail trade/finance and Insurance/construction/education, learning support/industries unable to classify/information and communications/government, except elsewhere classified/scientific research, professional and technical services/living-related and personal services and amusement services/real estate, goods rental and leasing/transport and postal service/accommodations, eating and drinking services/electricity, gas, heat supply, and water/agriculture and forestry, fisheries/mining and quarrying of stone and gravel), working status (full-time, part-time, self-employed), workplace size (<50 workers/50–100 workers/100–300 workers/300–1,000 workers/>1,000 workers), and labor characteristics (daily average hours of working, weekly average hours of working, daily average hours of standing, number of times required to carry heavy objects, number of night shifts in the previous month).

### 2.4 Statistical analysis

Based on the Bernoulli distribution, we divided the participants into two groups for exploratory factor analysis (EFA) and confirmatory factor analysis (CFA), respectively. We determined the number of factors based on a scree plot and Kaiser criterion (eigenvalue >1). We performed EFA based on maximum likelihood estimation with Promax oblique rotation. The items with factor loadings <0.5 or items that were heavily loaded with two or more items were discarded. To determine the internal consistency of the items, we developed a final model and computed the item test, item-rest correlation, and Cronbach’s alpha coefficients. Once the final model was determined using EFA, we performed consecutive CFA and computed the fit indices and factor loadings to confirm the best-fitting model with a comparative fit index (CFI) >0.9, Tucker–Leis index (TLI) >0.95, and root mean square residual (RMSR) <0.08. We created a best-fit model until the lowest number of Akaike information criterion statistics was reached.

Next, we computed a correlation coefficient with the total score of the MSQ (a menopause patient-reported outcome [17]) for criterion validity and the Copenhagen Burnout Inventory (CBI; a validated psychologically worn-out scale [20]) for concurrent validity. Third, each factor of the disability index was divided into a binary at its median based on the summation of the recorded response patterns (i.e., the upper half or lower half), with a higher score indicating higher disability. The chi-square test and logistic regression models were used to investigate whether the upper half of each factor was associated with an increased risk of work absenteeism. We computed odds ratios (OR) along with 95% confidence intervals (CI) after adjusting for age, body mass index, socioeconomic variables of educational attainment, marital status, presence of a child, working status, and Copenhagen burnout binary variable divided by median. Finally, to estimate the risk of absenteeism, crude receiver operating characteristic (ROC) curves were drawn with an area under the curve (AUC) calculation, which is an effective measure of accuracy and AUC values of 0.6–0.8 are considered acceptable for the prediction of absenteeism based on the Hosmer and Lemeshow test [21]. To determine the optimal total score of the index, we calculated the Youden index as sensitivity + specificity − 1, which indicates the maximum vertical distance of the ROC curve from a point (x, y) on the diagonal line, and thus maximizes the difference between the true-positive and false-positive fractions. We calculated the optimal cutoff point of the index for absenteeism corresponding to the largest Youden index value with a lower boundary of 95%CI of AUC >0.6.

All analyses were performed using SAS (version 9.4, SAS Institute Inc., Cary, NC, USA) except for ROC curve that was drawn by Stata version 17 (Stata Corp., College Station, TX, USA). The significance level was determined at 0.05 and was two-sided.

## 3. Results

Among the 3,645 participants (average age, 49.8 years) who answered the self-administered questionnaire, 57.8% had menstruation at the time of responding. Among 2,367 participants who answered that they had menstruated within a year, 19.6% (*n* = 464) had amenorrhea for more than 60 days and 24.5% (*n* = 626) had their menstrual cycle disrupted for more than seven days. Approximately 20% of women had used menstruation-related medicines—pain-killer (63.5%), hormone replacement therapy (17%), Chinese herbal medicines (12%), or supplements (5.3%).

Based on occupation, nearly half (49.9%) of the participants were clerical workers, followed by service workers (16.5%) and professional and engineering workers (14.6%) (Table 1). In the industrial classification, most women were into medical, healthcare, and welfare (15.7%) and manufacturing jobs (15.5%), followed by those who were in compound services (11.2%) or wholesale and retail trade (10.3%) (Supplementary table 1).

**Table 1:** Characteristics of participants (n = 3645)

	**N or Mean**	**% or SD**
Age, mean ± SD	49.8	±3.3 years
Body Mass Index, mean ± SD	21.4	±3.7 kg/m^2^
Marital status		
Single	2132	58.5
Married	1513	41.5
Children		
(+)	1512	41.5
(−)	2133	58.5
Annual household income		
>8 million JPY	901	24.7
6–8 million JPY	626	17.2
4–6 million JPY	824	22.6
2–4 million JPY	1012	27.8
<2 million JPY	282	7.7
Education attainment		
High/junior/elementary school	1104	30.3
2 year-college	1354	37.2
University/Graduated	1187	32.6
Occupation		
Clerical workers	1820	49.9
Service workers	601	16.5
Professional and engineering workers	533	14.6
Sales workers	175	4.8
Manufacturing process workers	132	3.6
Administrative and managerial workers	122	3.4
Others	262	7.2
Industry		
Medical, Health Care and Welfare	571	15.7
Manufacturing	565	15.5
Compound Services	407	11.2
Wholesale and Retail trade	375	10.3
Finance and Insurance	262	7.2
Construction	241	6.6
Education, Learning Support	189	5.2
Others	1035	28.4

Overall, 77% of the participants, including self-employed women, worked full-time, and 41.8% worked in small workplaces with <50 employees. In response to questions on work productivity, 241 women (6.6%) reported that they had been absent from work due to menstrual symptoms during the perimenopausal period (Table 2). Of these, nearly half (*n* = 133) reported that the median total absence period was one day per week, and 61 women (one-fourth) reported a median of two months per year, which seriously decreased work productivity (Table 2).

**Table 2:** Working status, workplace size, labour characteristics, Copenhagen burnout scale, and work productivity.

		**N or median**	**% or IQR**
Working status			
Full-time worker		2421	66.4
Part-time worker		821	22.5
Self-employed		403	11.1
Workplace size			
<50 workers		1524	41.81
50–100 workers		364	10
100–300 workers		486	13.3
300–1,000 workers		431	11.8
>1,000 workers		840	23.1

Labour characteristics			
Daily average hours of working, median (IQR)		8	(7, 8)
Weekly average hours of working, median (IQR)		40	(25, 42)
Daily average hours of standing, median (IQR)		2	(1, 5)
Numbers of carrying heavy objects, N (%)		1036	28.4
Numbers of a night shift in previous month, N (%)		335	9.2

Copenhagen Burnout Inventory, median (IQR)			
Personal Burnout		21	(4–46)
Work related Burnout		36	(21–50)
Client related Burnout		38	(21–54)
Work productivity			
Ever been absent due to menstrual symptoms			
	Yes	241	6.6
	No	3113	85.4
	Do not remember	291	8.0
How long total absence at maximum (n = 241)			
	Reported n = 61	2 (1, 6)	month/year
	Reported n = 21	1 (1, 2)	week/month
	Reported n = 133	1 (1, 2)	day/week
	Reported n = 26	2 (1, 6)	hr/day

IQR indicates “interquartile range.”

The initial solution based on a scree plot revealed four factors with eigenvalues of 1.0 or greater. Using the split-half sample, the EFA was performed with loadings <0.5, to shorten the scale. After excluding items that either cross-loaded or did not load, we extracted 21 items with a four-factor solution (Table 3), accounting for 89% of the total variance. The first factor contained eight items and was labeled “Psychological symptoms (Cronbach’s α = 0.955).” This domain included depression, anxiety, fatigue, and lack of interest in work. The second factor contained six items and was labeled “Physical symptoms (Cronbach’s α = 0.868).” This domain included digestive and respiratory symptoms and abnormal sensations. The third factor contained four items and was labeled “Sleep difficulty (Cronbach’s α = 0.919).” This domain included difficulties in the induction and maintenance of sleep. The fourth factor contained three items and was labeled “Human relationship (Cronbach’s α = 0.918).” Cronbach’s alpha values for each factor were high (>0.8), and the item correlations between each factor were reasonably high, suggesting good reliability and high internal consistency for each factor (Table 3). Using the split-half sample for the CFA, excellent model fit indices for the four-factor solution were confirmed (RMSR = 0.079, TLI = 0.929, CFI = 0.938).

**Table 3:**
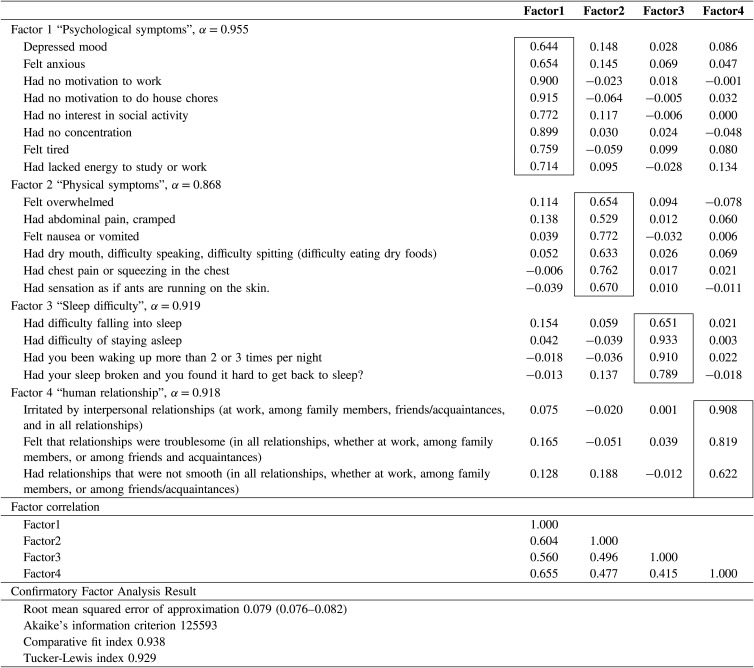
Results of Exploratory and Confirmatory Factor Analyses

Based on Factor Analyses with Maximum likelihood and Promax rotation.

The correlation coefficients between each factor as well as the total score and the gold standard of menopause were >0.74 at their minimum, suggesting high criterion validity (Table 4). The correlation coefficients with the three subscales of the Copenhagen Burnout Inventory were >0.405 at their minimum, suggesting high concurrent validity (Table 4).

**Table 4:** Correlation between the developed scale and the widely used menopause checklist and Copenhagen burnout (n = 3645)

**Factor**	**Factor 1**	**Factor 2**	**Factor 3**	**Factor 4**	**Total score ** **(max 147 points)**
**Domain name**	**Psychological symptoms**	**Physiological symptoms**	**Sleep difficulty**	**Human relationship**	
# of item	8	6	4	3	21
median (IQR)					
	20 (11, 32)	9 (6, 13)	9 (4, 17)	6 (3, 12)	49 (30, 72)

Correlation coefficient					
Total score of menopause checklist					
	0.873*	0.741*	0.755*	0.768*	0.932*
Copenhagen Burnout Inventory, median (IQR)					
Personal Burnout					
	0.700*	0.545*	0.498*	0.635*	0.716*
Work-Related Burnout					
	0.634*	0.497*	0.426*	0.575*	0.641*
Client related Burnout					
	0.590*	0.459*	0.405*	0.603*	0.612

Based on Spearman Correlation Coefficient

*P < 0.0001

IQR indicates “inter quartile range”.

Women who experienced absenteeism due to menopause-related symptoms tended to have a higher level of binary score for each factor as well as the total score (*p* < 0.0001 for all factors, Table 5). Multivariate logistic regression models demonstrated that a higher level of binary score for each factor was associated with an increased risk of work absenteeism after adjusting for age, body mass index (BMI), educational attainment, marital status, presence of a child, working status, and the Copenhagen burnout binary variable divided by the median (Table 5).

**Table 5:** Validation of the developed scale for work absenteeism

	**Work absenteeism experience due to menopause related symptoms**					**Work productivity absenteeism**			

	**Yes**		**No / Do not remember**		**p-value**	**Crude odds ratio**	**Adjusted odds ratio^a^**		
	
	**n = 241, 6.6%**		**n = 3404, 93.4%**				**PBO model**	**WBO model**	**CBO model**
Factor 1	n	%	n	%	<0.0001				
Upper half	197	10.6	1658	89.4		4.72 (95%CI:3.38–6.58)	3.75 (95%CI:2.58–5.45)	4.27 (95%CI:2.96–6.17)	3.95 (95%CI:2.76–5.67)
Lower half	44	2.5	1746	97.5		1	1	1	1
Factor 2					<0.0001				
Upper half	190	10.8	1573	89.2		4.33 (95%CI:3.16–5.95)	3.43 (95%CI:2.45–4.79)	3.77 (95%CI:2.70–5.27)	3.63 (95%CI:2.61–5.04)
Lower half	51	2.7	1831	97.3		1	1	1	1
Factor 3					<0.0001				
Upper half	187	9.7	1744	90.3		3.30 (95%CI:2.42–4.49)	2.58 (95%CI:1.86–3.57)	2.82 (95%CI:2.03–3.91)	2.74 (95%CI:1.98–3.79)
Lower half	54	3.2	1660	96.9		1	1	1	1
Factor 4					<0.0001				
Upper half	173	9.9	1568	90.1		2.98 (95%CI:2.23–3.98)	2.14 (95%CI:1.55–2.95)	2.44 (95%CI:1.77–3.35)	2.30 (95%CI:1.66–3.17)
Lower half	68	3.6	1836	96.4		1	1	1	1
Total score					<0.0001				
Upper half	201	10.8	1656	89.2		5.30 (95%CI:3.75–7.50)	4.41 (95%CI:2.99–6.51)	5.07 (95%CI:3.46–7.44)	4.61 (95%CI:3.17–6.71)
Lower half	40	2.2	1748	97.8		1	1	1	1

^a^Adjusted for age, BMI, educational attainment, marital status, presence of a child, working status, and Copenhagen burnout binary variable divided by median.

Based on a multivariable logistic regression models

For Copenhagen burnout inventory, we put PBC, CBO, and WBO alternatively in to each model.

**Fig. 1:**
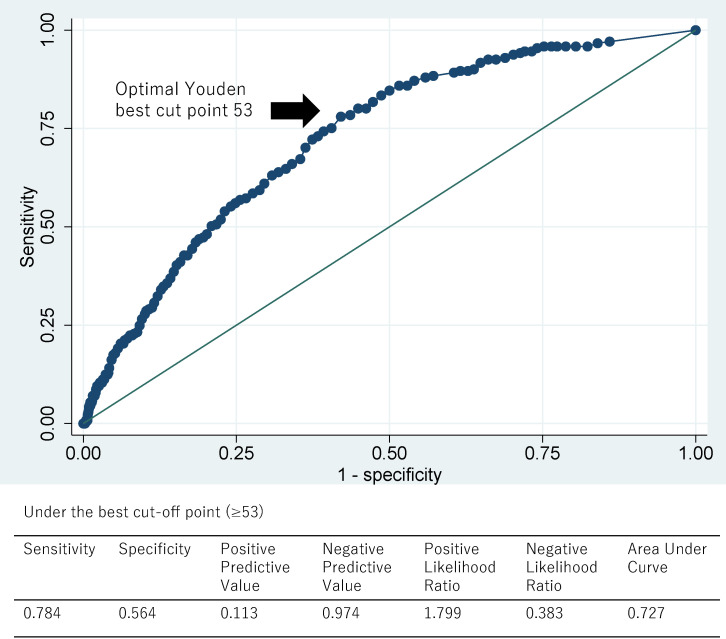
Receiver operating characteristic (ROC) curves of the disability index to estimate the risk of absenteeism Based on the largest Youden index, 53 points of the disability index revealed 78% sensitivity, 58% specificity, and an AUC of 0.727 (95%CI: 0.696–0.757), which was considered acceptable, referring to Hosmer and Lemeshow test.

Based on the largest Youden index, we estimated the optimal cut-off point as 53 points of the disability index, with 78% sensitivity, 58% specificity, and an AUC of 0.727 (95%CI: 0.696–0.757, Fig. 1), which was considered acceptable, referring to Hosmer and Lemeshow test [21]. Other efficacy indicators including positive and negative predictive values (PPV and NPV) and likelihood ratios are also shown in Fig. 1.

## 4. Discussion

In this study, we developed a disability index scale for working women in perimenopausal period based on responses from 3645 female workers aged 45–56 years, regardless of ethnic background, Based on factor analyses, we were able to extract various menopause-related symptoms including the four factors with 21 items: psychological symptoms (eight items), physiological symptoms (six items), sleep difficulty (four items), and human relationship (three items). Exhibiting high Cronbach’s alpha and correlation coefficients with the menopause diagnostic scale (i.e., MSQ) and the burnout subscales (i.e., CBI), the Women’s Disability Index developed in this study has a high degree of reliability, and criterion and concurrent validity. Additionally, a higher level of each factor of the disability index was significantly associated with work absenteeism. The ROC curve of the disability index revealed 78% sensitivity, 58% specificity with an AUC of 0.727 under the best cut-off point, suggesting that this index has a high predictability for absenteeism. This study provides a work productivity indicator for women in menopausal period that can be useful for labor management in the workplace. We discuss further, considering the strengths and limitations of the study.

Our scale was able to include important aspects of work including human relationships in the workplace, as a measure of menopausal disability. Irritability and other anxiety symptoms associated with menopause may affect existing relationships, whereas depression may interfere with building new relationships. Menopausal symptoms can lead to decreased social desirability and feelings of embarrassment, with unwelcome comments from coworkers [14, 22]. Women may feel ashamed and afraid to bring up their symptoms for fear of denial or stigma that it will undermine others’ perception of them at work or affect their chances of promotion [23]. Work-related stress, fatigue and career conflicts also affect the experiences of women in their midlives [24]. In the workplace, recognition of these symptoms and their impact on work productivity is useful for managers and occupational health staff to provide adequate support to such women.

Notably, our developed scale which was able to predict absenteeism among working women, can be used, not only by female workers as a self-care evaluation tool but also by line managers or employers as a labor management tool. Previous literatures [25, 26] report that menstrual symptoms cause lower perceived general health, intention to leave the workplace and overall poor presenteeism also leading to work productivity loss. Thus, the developed scale may be useful to identify high-risk individuals for subsequent failure to achieve career advancement opportunities, career decline, or even early retirement.

This study’s strength is that the subjects’ nationality was not restricted only to Japanese. Thus, the results of this study can help promote women’s employment in a country like Japan, where the population is aging and the workforce is declining [27]. Other strengths include a large sample size of female workers in the perimenopausal period and a sequential analytic scheme including face validity of initial items, EFA and CFA, with high validity and reliability. For face validity, our task team, which included relevant specialists and female workers, created 76 items with a high level of validity. However, this study has several limitations that should be addressed. First, the generalizability of our study may be limited. According to 2013 Labour Force Survey in Japan [28] “Medical care and welfare” was the highest (20.5%), followed by “Wholesale and retail trade” (20.0%) and “Manufacturing” (11.4%). In contrast, our study demonstrated that “Medical care and welfare” (15.7%) and “Manufacturing” (15.5%) was the highest, followed by “Compound Services” (11.2%) and “Wholesale and retail trade” (10.3%). For the Japan Standard Occupational Classification [29], “clerical workers” accounted for a conspicuously high 27.2%, followed by “service workers” (19.5%) and “professional/technical workers” (17.2%). In contrast, our study demonstrated that Clerical workers consists 49.9%, followed by “service workers” (16.5%) and “professional/technical workers” (14.6%). These differences between our study and the Labour survey may be due to the recruitment method being based on internet. In addition, considering the average 8 hours of work per day and night shift ratio of less than 10%, we can infer that majority of our target population is a group of physically unburdened office workers. This may have influenced the severity of menopause-related symptoms. Second, although our participants included any nationality, the response requires at least minimum levels of Japanese language reading and understanding ability, and therefore, we can assume that the majority of respondents may be Japanese or Asian women. Nevertheless, if the response rate differs according to ethnicity or culture, the result of our study may have been influenced. Similarly, the initial 76 items included “ordering meals on Uber Eats” which is a recent new food ordering system and may not be applicable to all participants. These items were initially discarded due to small factor loading. Third, there were several items that were not retained in our final model but are relevant to menopause symptomatology. For example, vasomotor symptoms, including hot flushes and night sweats, which are the most common menopausal symptoms [30]. These relevant items were cross-loaded over two factors of sleep and other physical symptoms in our factor analyses. One of the possible reasons for the cross-loading may have been due to the influence of lifestyle factors, such as alcohol, caffeine, and smoking [31], that could cause greater vasomotor symptoms, which might further cause sleep disturbances [32]. Other symptoms like vaginal dryness, decreased libido, and urinary incontinence are also associated with menopause [33], however the frequency of responses was extremely low which may have resulted in low factor loading in factor analysis.

Fourth, we investigated predictability for absenteeism only. However, future studies should be conducted to predict presenteeism. In addition, predictability investigated in our study is based on a cross-sectional time frame. Future studies are warranted to prove longitudinal predictability by using cohort study design. Fifth, our developed disability index is highly reliable and valid. However, it should be noted that PPV is low at 11%. This indicates that the usefulness of this index would be limited in general female workers population but increases with high-risk of absenteeism among menopausal women just as PPV increases with high prevalence of target disease [34]. With these limitations, our results should be interpreted with caution.

## Conclusion

Despite the aforementioned limitations, we developed a perimenopausal women disability index, consisting of four factors with 21 items, with high validity and reliability. This index can be used as a significant indicator for work absenteeism among female workers and utilized to measure the overall health of working women in the workplace by female workers themselves and by employers.

## Supplementary information

**Additional file 1:** **Supplementary table 1.** The classification of occupation and industry.

## Declarations

### Availability of data and materials

The datasets used and/or analyzed during the present study are available from the corresponding author on reasonable request.

## Appendix

“During the period when your menstrual cycle began to change (short, low volume, irregular, etc.) or in the years before and after menopause (if you are not sure, please answer with your current symptoms), how often did you have any of the following symptoms?”

1. Mood depression
2. Anxiety
3. Feeling of extreme psychological instability (sudden loneliness or becoming tearful)
4. Irritability
5. Lack of motivation to work
6. Lack of motivation to perform house chores
7. Lack of interest in social activities
8. Lack of concentration
9. Tiredness
10. Gain or loss of appetite
11. Difficulty in falling asleep
12. Hard to stay asleep
13. Waking up from sleep more than 2 or 3 times per night
14. Difficulty in resuming sleep
15. Getting awake due to hot flushes and sweating
16. Overwhelmed feeling
17. Breast pain, tension, or tenderness
18. Weight gain
19. Feeling of worthlessness
20. Loss of hair moisture or dry hair
21. Dry, uncomfortable, and tired eyes
22. Stomach upset/heartburn
23. Feeling that the world would be better off without you
24. Lacked energy to study or work
25. Increased shoulder or neck pain
26. Inability to clear head, forgetfulness, difficulty in thinking clearly
27. Lack of self-confidence
28. Headache
29. Skin trouble
30. Difficultly in breathing
31. Abdominal pain or cramps
32. Dizziness
33. Tension
34. Aversion to socializing
35. Sweating
36. Inability to judge
37. Stumbling or falling over
38. Nausea or vomiting
39. Dry mouth, difficulty in speaking, spitting, or eating dry foods
40. Hot flushes on face or upper body
41. Chest pain or squeezing in the chest
42. Tinnitus
43. Edema on face, legs, or fingers
44. Broken dishes or cut finger easily
45. Back pain
46. Concern about trivial things
47. Heart beating or pounding
48. Difficulty in remembering things, forgetfulness
49. Reduced working and learning ability
50. Increased sensitivity to noise
51. Numbness in the limbs
52. Chilly lower back or limbs
53. Difficulty in seeing (e.g., blurred vision or invisible areas)
54. Diarrhea or constipation
55. Abdominal distention
56. Pain in finger or knee joint
57. Muscle pain (cramps in the legs)
58. Sensation akin to that of ants running on the skin
59. Frequent urination
60. Urine discharge while sneezing, sniffling, or laughing out loudly
61. Interest in sexual activity
62. Decreased frequency of sexual activities
63. Pain during sexual activity
64. Expending more time on routine work
65. Feeing of lower accomplishment at work
66. Increased careless mistakes at work
67. Instances of late arrival, early departure, or was absence from work
68. Expending more time in finishing usual household chores
69. Inability to cook for self
70. Ordering meals, for example, on Uber Eats, or buying ready-made food
71. Inability to clean a room
72. Inability to wash clothes
73. Feeling of irritation in interpersonal relationships (at work, with family members, friends or acquaintances, and in all other relationships)
74. Feeling that relationships are troublesome (at work, with family members, friends, or acquaintances, and in all other relationships)
75. Relationship conflicts over trivial matters (at work, with family members, friends, or acquaintances, and in all other relationships)
76. Tumultuous relationships (at work, with family members, friends, or acquaintances, and in all other relationships)
