# Supplementary material for: Scale development and validation of perimenopausal women disability index in the workplace
Source: Environ Health Prev Med. 2024 Feb 2;29:4. doi: 10.1265/ehpm.23-00239 (PMC10853391; doi:10.1265/ehpm.23-00239)
Supplement: Supplementary file 1 — Additional file 1: Supplementary table 1. The classification of occupation and industry. [file ehpm-29-004-s001.docx]

**Supplementary table 1. The classification of occupation and industry.**

| Occupational classification | | N | % |
| --- | --- | --- | --- |
|  | Clerical workers | 1820 | 49.93 |
|  | Service workers | 601 | 16.49 |
|  | Professional and engineering workers | 533 | 14.62 |
|  | Workers not classifiable by occupation | 190 | 5.21 |
|  | Sales workers | 175 | 4.8 |
|  | Manufacturing process workers | 132 | 3.62 |
|  | Administrative and managerial workers | 122 | 3.35 |
|  | Transport and machine operation workers | 21 | 0.58 |
|  | Construction and mining workers | 19 | 0.52 |
|  | Agriculture, forestry and fishery workers | 13 | 0.36 |
|  | Carrying, cleaning, packaging, and related workers | 13 | 0.36 |
|  | Security workers | 6 | 0.16 |
| Industrial classification | | N | % |
|  | Medical, Health Care and Welfare | 571 | 15.67 |
|  | Manufacturing | 565 | 15.5 |
|  | Compound Services | 407 | 11.17 |
|  | Wholesale and Retail trade | 375 | 10.29 |
|  | Finance and Insurance | 262 | 7.19 |
|  | Construction | 241 | 6.61 |
|  | Education, Learning Support | 189 | 5.19 |
|  | Industries unable to classify | 179 | 4.91 |
|  | Information and Communications | 162 | 4.44 |
|  | Government, Except Elsewhere Classified | 156 | 4.28 |
|  | Scientific Research, Professional and Technical Services | 118 | 3.24 |
|  | Living-Related and Personal Services and Amusement Services | 110 | 3.02 |
|  | Real Estate, Goods Rental and Leasing | 101 | 2.77 |
|  | Transport and Postal Service | 88 | 2.41 |
|  | Accommodations, Eating and Drinking Services | 65 | 1.78 |
|  | Electricity, Gas, heat supply and water | 31 | 0.85 |
|  | Agriculture and Forestry, Fisheries | 21 | 0.58 |
|  | Mining and Quarrying of stone and Gravel | 4 | 0.11 |
